# KK-DBP: A Multi-Feature Fusion Method for DNA-Binding Protein Identification Based on Random Forest

**DOI:** 10.3389/fgene.2021.811158

**Published:** 2021-11-29

**Authors:** Yuran Jia, Shan Huang, Tianjiao Zhang

**Affiliations:** ^1^ College of Information and Computer Engineering, Northeast Forestry University, Harbin, China; ^2^ Department of Neurology, The Second Affiliated Hospital of Harbin Medical University, Harbin, China

**Keywords:** DNA-binding protein, position specificity score matrix, random forest, feature extraction, multi-feature fusion

## Abstract

DNA-binding protein (DBP) is a protein with a special DNA binding domain that is associated with many important molecular biological mechanisms. Rapid development of computational methods has made it possible to predict DBP on a large scale; however, existing methods do not fully integrate DBP-related features, resulting in rough prediction results. In this article, we develop a DNA-binding protein identification method called KK-DBP. To improve prediction accuracy, we propose a feature extraction method that fuses multiple PSSM features. The experimental results show a prediction accuracy on the independent test dataset PDB186 of 81.22%, which is the highest of all existing methods.

## Introduction

Proteins are spatially structured substances formed by the complex folding of amino acids into polypeptide chains through dehydration and condensation. Proteins are the material basis of life and they are required for every vital activity. Given the vast number of proteins and their roles, protein classification has always been central to the study of proteomics. DNA-binding proteins (DBP) are a very specific class of proteins whose specific binding to DNA guarantees the accuracy of biological processes and whose nonspecific binding to DNA guarantees the high efficiency of biological processes (Gao et al., 2008). DNA-protein interactions, such as gene expression and transcriptional regulation, occur ubiquitously throughout the biological activities of living bodies (Liu et al., 2019; Shen and Zou, 2020; Xu et al., 2021a). All of these interactions are tightly linked to DBP, where the fraction of DNA-binding proteins in eukaryotic genes is approximately 6–7%.

The role of DBP in biological activities has gained a lot of attention in recent years, as various large genome projects and research on DBP identification have rapidly progressed. However, identifying DBP using traditional biochemical analyses is inefficient and expensive (Li and Li, 2012; Xu et al., 2021b). In recent years, machine learning methods have been widely used in the field of bioinformatics (Jiang et al., 2013; Geete and Pandey, 2020; Tao et al., 2020; Wang et al., 2021a; Long et al., 2021). Using machine learning methods for DNA-binding protein identification can enable rapid and accurate prediction of DBP from a large number of proteins, while drastically reducing prediction costs (Fu et al., 2018). Because the number of proteins is large and promiscuous, overcoming every classification prediction problem with one method is difficult, if not impossible (Wang et al., 2021b). Therefore, we must continue to propose effective methods for high-quality DBP prediction and identification in order to understand the significance of more vital activities and to promote further progress within the bioinformatics field.

Feature extraction methods can be broadly classified into two categories: those based on structural information and those based on sequence information (Kim et al., 2004; Meng and Kurgan, 2016; Qu et al., 2019; Ao et al., 2021a; Lv et al., 2021a; Liu et al., 2021; Tang et al., 2021; Wu and Yu, 2021); (Stawiski et al., 2003) proposed a model based on protein structure that utilises a neural network approach incorporating information like residue and hydrogen bond potential. Liu et al. (Liu et al., 2014) developed a model called IDNA-prot|dis, based on the pseudo amino acid composition (PseAAC) of protein sequence information. iDNAPro-PseAAC (Liu et al., 2015), which uses a similar feature extraction method, adopts a prediction model based on a support vector machine to predict DBP. IDNA-prot (Lin et al., 2011) was constructed based on physicochemical properties and random forest (RF) classification. In addition, a support vector machine model based on k-mer and autocovariance transformation was proposed by Dong et al. (Liu et al., 2016). Local-DPP (Wei et al., 2017a) used random forests based on PSE-PSSM features to predict DBP. MK-FSVM-SVDD is a multiple kernel SVM prediction tool based on the heuristic kernel alignment developed by Ding et al. (Zou et al., 2021) to identify DBP. In addition, two models for predicting DBP were developed: DNA-prot (Kumar et al., 2009) and DNAbinder (Kumar et al., 2007). Lu et al. (Lu et al., 2020) developed a prediction model for DBP based on support vector machines using Chou’s five-step rule.

Currently, a number of DNA-binding protein prediction methods based on different strategies exist. Unfortunately, most of these DBP prediction methods fail to extract features based on evolutionary information, so their robustness and prediction accuracy have much room for improvement. To address these issues, more research is needed with regard to feature extraction and the selection of classifiers (Zuo et al., 2017; Zheng et al., 2019).

**FIGURE 1:**
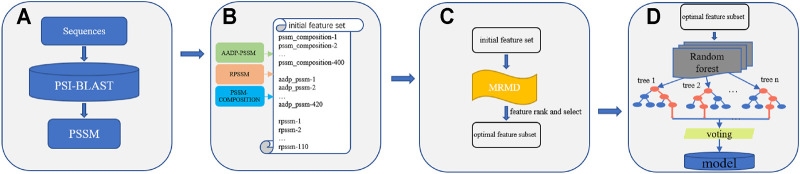
Framework of KK-DBP. **Step A**: Construction of Position Specificity Score Matrices for protein sequences. **Step B:** Extraction of three features: AADP-PSSM, PSSM-COMPOSITION, and RPSSM as the initial feature set for a single sample. **Step C:** Feature ranking and selection using the MRMD algorithm. **Step D:** Identification of DBP using random forests.

In this paper, we propose a new DNA-binding protein prediction method called KK-DBP. We first obtained the position specificity score matrix (PSSM) of the protein sequence for each sample used to train the model. PSSM information was then used to extract three features of each sample: PSSM-COMPOSITION (Zou et al., 2013), RPSSM (Ding et al., 2014) and AADP-PSSM (Liu et al., 2010), which were combined to form the initial feature set of each sample. The final initial feature set of each sample reached 930 dimensions. To avoid feature redundancy and improve prediction accuracy, KK-DBP used the max relevance max distance (MRMD) (Zou et al., 2016) feature ordering method to establish the optimal feature subset for model training. Finally, a new DBP prediction model was constructed using the random forest learning method. The complete method framework is shown in Figure 1:

## Materials and Methods

### Dataset

The dataset is one of the key factors determining the quality of the predictive model and is the cornerstone of machine learning algorithm learning, which directly affects the final effect of the model, so dataset construction is meticulous and complex (Liang et al., 2017; Su et al., 2021). Other researchers have proposed many prediction models for DNA-binding proteins that have been pertinent to objectively comparing existing data. In the present study, we have used protein sequences from the PDB database as our training dataset and test dataset. Table 1 shows the contents of the dataset:

**TABLE 1:** benchmark datasets used in this paper.

Data set	PDB1075	PDB186
Positive	525	93
Negative	550	93
Total	1075	186

The training set PDB1075 contained 525 DNA-binding proteins and 550 non-DNA-binding proteins, and the test set PDB186 contained 93 DNA-binding proteins and 93 non-DNA-binding proteins. The dataset construction rules are as follows:

$$S=S^{+}\cup S^{-}$$ (1)

where $S^{+}$ is the positive subset containing only DNA-binding proteins, and $S^{-}$ is the negative subset containing only non-DNA-binding proteins.

### Feature Extraction

Feature extraction is very important to modeling sequence classifications, which directly affect the accuracy of predictive models (Zhang et al., 2020a; Lv et al., 2021b). Evolutionary information is among the most important information we have regarding protein function and genetics (Zuo et al., 2014). Position specificity score matrices (PSSM) can intuitively display protein evolutionary information. Thus, the feature extraction method based on PSSM is widely used in protein classification.

#### Position specificity Score Matrices

In 1997, Altschul et al. (Altschul et al., 1990) proposed the BLAST algorithm. When given a protein sequence, BLAST can represent the evolutionary information of a protein by aligning it with data in a specific database and extracting a position specific score matrix (PSSM). To improve the prediction accuracy of proteins, our method predominantly utilises protein evolution information to extract features. For the training and test sets used in our method, the PSSM matrices for each sequence were generated by three PSI-BLAST iterations with an E-value of 0.001. The PSSM is a matrix of size L × 20, where L is the length of the protein sequence and 20 is the number of amino acids. Coordinates (i, j) in the position specificity score matrix. (PSSM) represent the log score for the amino acid at position i being replaced by the log score of the amino acid at position j. When the coordinate value is greater than 0, it indicates that during the alignment, there is as large probability that the amino acid at the corresponding position in the sequence is mutated to 20 native amino acids. The higher the value is when the number is a negative integer, the less prone it is to alteration. This numerical pattern indicates the probability of the mutation of a residue in a given protein sequences. Its matrix form behaves as follows:

$$PSSM_{L\times 20}=\left[\begin{array}{cccc} p_{1,1} & p_{1,2} & \cdots & p_{1,20}\\ p_{2,1} & p_{2,2} & \cdots & p_{2,20}\\ \vdots & \vdots & \cdots & \vdots \\ p_{L,1} & p_{L,2} & \cdots & p_{L,20} \end{array}\right]$$ (2)

#### Reduced Position Specificity Score Matrices and Position Specificity Score Matrices-Composition

PSSM-COMPOSITION is generated by adding the same amino acid rows in the original PSSM matrix, dividing by the sequence length and scaling to [-1,1]. For each protein sequence PSSM matrix, a 400-dimensional vector feature $\left\{d_{1},d_{2},d_{3},...,d_{400}\right\}$ is generated.

Li et al. (Li et al., 2003) first proposed that 10 might be the minimum number of residue types (letters) needed to construct a reasonably folded model. Reduced PSSM (RPSSM) borrowed this idea and simplified the original PSSM matrix with form L × 20 to one with form L × 10.

$a_{1}a_{2}\ldots a_{L}$ is a protein in the dataset, $a_{i}$ is assumed to be mutated to s, and $p_{i,s}$ represents the pseudo composition component of amino acid $a_{i}$ . The pseudo composition of all amino acids in protein $a_{1}a_{2}\ldots a_{L}$ is defined as:

$$\;D_{s}=\frac{1}{L}\overset{L}{\underset{i=1}{\sum }}{\left(p_{i,s}-\frac{1}{L}\overset{L}{\underset{i=1}{\sum }}p_{i,s}\right)}^{2}\;\begin{array}{cc} s=1,2,...10; & i=1,2,...,L \end{array}$$ (3)

The dipeptide composition was later incorporated into the RPSSM method in order to overcome its inability to extract full sequence information. Assuming that $a_{i+1}$ is replaced by ‘t', the dipeptide pseudocomposition of $a_{i}a_{i+1}$ is defined as:

$$x_{i,i+1}=\frac{{\left(p_{i,s}+p_{i+1,t}\right)}^{2}}{2}\;\;\;\;\;s,t=1,2,\ldots 10;\;i=1,2,\ldots ,L-1$$ (4)

where $x_{i,i+1}$ represents the difference of $p_{i,s}$ and $p_{i+1,t}$ from their mean values. Finally, because each protein sequence in the dataset will consist of the pseudo composition of all of its dipeptides, we can generate a 110-dimensional vector feature of RPSSM, defined as follows:

$$\;\;\;\;\;\;\;D_{s,t}=\frac{1}{L-1}\overset{L-1}{\underset{i=1}{\sum }}x_{i,i+1}=\;\frac{1}{L-1}\overset{L-1}{\underset{i=1}{\sum }}\frac{{\left(p_{i,s}+p_{i+1,t}\right)}^{2}}{2}\;\;\;\;\;\;\;s,t=1,2,\ldots 10$$ (5)

#### AADP-Position Specificity Score Matrices

A protein’s structure is closely related to its amino acid composition. For every amino acid sequence in the dataset, AADP-PSSM produces a vector with dimensions 20 + 400 = 420. AADP-PSSM is divided into two parts. The amino acid composition is first extracted from its PSSM matrix: the average value of the PSSM matrix column of length 20 is called AAC-PSSM, where $x_{i}$ is the type of amino acid in the PSSM matrix and represents the average fraction of amino acid mutations during evolution. It is defined as follows:

$$x_{j}=\frac{1}{L}\overset{L}{\underset{i=1}{\sum }}p_{i,j}\;\left(j=1,2,\ldots ,20\right)\;$$ (6)

The traditional dipeptide composition was later extended to PSSM and represented with DPC-PSSM to avoid the loss of information due to an X in the protein, which was defined as a vector of 400 dimensions:

$$\begin{array}{cc} y_{i,j}=\frac{1}{L-1}\overset{L-1}{\underset{K=1}{\sum }}P_{k,i}\times P_{k+1,j} & \left(1\leq i,j\leq 20\right) \end{array}$$ (7)

#### Feature Selection

Feature redundancy or dimensionality disasters often occur during feature extraction. Feature selection not only reduces the risk of overfitting but also improves the model’s generalization ability and computational efficiency (Guo et al., 2020; Yang et al., 2021a; Ao et al., 2021b; Zhao et al., 2021). In the present paper, we use the max relevance max distance (MRMD) feature selection method to reduce the dimensions of the initial feature set (He et al., 2020).

In MRMD, feature selection is based primarily on the correlation between the subset and the target vector and the redundancy of the subset. When measuring correlations, MRMD used the Pearson correlation coefficient, which is defined as:

$$PCC\left(\vec{X},\vec{Y}\right)=\frac{\frac{1}{N-1}\sum_{k=1}^{N}\left(x_{k}-\frac{1}{N}\sum_{k=1}^{N}x_{k}\right)\left(y_{k}-\frac{1}{N}\sum_{k=1}^{N}y_{k}\right)}{\sqrt{\frac{1}{N-1}\sum_{k=1}^{N}{\left(x_{k}-\frac{1}{N}\sum_{k=1}^{N}x_{k}\right)}^{2}}\sqrt{\frac{1}{N-1}\sum_{k=1}^{N}{\left(y_{k}-\frac{1}{N}\sum_{k=1}^{N}y_{k}\right)}^{2}}}$$ (8)

where $\vec{X}$ and $\vec{Y}$ are two vectors, $x_{k}$ and $y_{k}$ are the *k*th elements in $\vec{X}$ and $\vec{Y}$ , and N is the total sample number. The initial feature set constructed using this method is $F=\left\{f_{1},f_{2},f_{3},\ldots ,f_{930}\right\}$ . The maximum correlation value $maxMR_{i}$ between feature $f_{i}$ and target class vector C is defined as:

$$maxMR_{i}=\left|PCC\left(\vec{f_{i}},\vec{C_{i}}\right)\right|\left(1\leq i\leq M\right)\;$$ (9)

where M is the initial feature set dimension, $\vec{f_{i}}$ is the vector composed of the *i*th feature of each instance, and $\vec{C_{i}}$ is the vector composed of the target category of each instance.

When evaluating the similarity between two vectors, MRMD uses the distance functions Euclidean distance (ED), cosine similarity (COS) and Tanimoto coefficient (TC) to measure:

$$ED\left(\vec{X},\vec{Y}\right)=\sqrt{\overset{N}{\underset{k=1}{\sum }}{\left(x_{k}-y_{k}\right)}^{2}}$$ (10)

$$COS\left(\vec{X},\vec{Y}\right)=\frac{\sum_{k=1}^{N}x_{k}y_{k}}{\sqrt{\sum_{k=1}^{N}x_{k}^{2}}\cdot \sqrt{\sum_{k=1}^{N}y_{k}^{2}}}$$ (11)

$$TC\left(\vec{X},\vec{Y}\right)=\frac{\sum_{k=1}^{N}x_{k}y_{k}}{\sum_{k=1}^{N}x_{k}^{2}+\sum_{k=1}^{N}y_{k}^{2}-\sum_{k=1}^{N}x_{k}y_{k}}$$ (12)

We use the mean of the three above as the maximum distance $maxMD_{i}$ for feature i:

$$ED_{i}=\frac{1}{M-1}\sum ED\left(\vec{f_{i}},\vec{f_{k}}\right)\left(1\leq k\leq M,k\neq i\right)$$ (13)

$$COS_{i}=\frac{1}{M-1}\sum COS\left(\vec{f_{i}},\vec{f_{k}}\right)\left(1\leq k\leq M,k\neq i\right)$$ (14)

$$TC_{i}=\frac{1}{M-1}\sum TC\left(\vec{f_{i}},\vec{f_{k}}\right)\;\left(1\leq k\leq M,k\neq i\right)$$ (15)

$$maxMD_{i}=\frac{1}{3}\left(ED_{i}+COS_{i}+TC_{i}\right)\;\left(1\leq i\leq M\right)$$ (16)

The MRMD values of all the features are calculated with the above two constraints. The PageRank algorithm is used to sort the initial feature set from high importance. One feature is added to the feature subset at a time and is used to train the model to determine which subset is the best.

#### Classification Algorithm

Protein prediction is usually described as a binary classification problem (Zhai et al., 2020; Zhang et al., 2021; Zulfiqar et al., 2021). We selected the random forest learning method for prediction modelling in the present study. Because the random forest method randomly extracts features and samples during construction of a decision tree set, it is more suitable to addressing the problem of high feature dimensions. By using RandomizedSearchCV and GridSearchCV for parameter selection, the random forest model constructed finally includes 800 subtrees, in which each tree has no limit, and a single decision tree is allowed to use all features. The maximum depth of each decision tree is 50.

## Results

### Measurements

We selected four different performance measures, accuracy (ACC), specificity (SP), sensitivity (SN) and Matthew’s correlation coefficient (MCC), to evaluate the methodology used by this study to demonstrate the predictive ability of the model used (Wei et al., 2014; Wei et al., 2017b; Manavalan et al., 2019a; Manavalan et al., 2019b; Jin et al., 2019; Su et al., 2019; Li et al., 2020a; Liu et al., 2020a; Ao et al., 2020; Li et al., 2020b; Zhang et al., 2020b; Yu et al., 2020; Zhao et al., 2020; Wang et al., 2021c; Zhu et al., 2021). The equations for determining these four parameters are shown below:

$$ACC=\frac{TN+TP}{TN+FP+FN+FP}\times 100\%$$ (17)

$$MCC=\frac{TP\times TN-FP\times FN}{\sqrt{\left(TP+FN\right)\times \left(TN+FN\right)\times \left(TP+FP\right)\times \left(TN+FP\right)}}$$ (18)

$$SN=\frac{TP}{TP+FN}\times 100\%$$ (19)

$$SP=\frac{TN}{TN+FP}\times 100\%$$ (20)

Where TP represents positive samples predicted to be positive by the model, FP represents negative samples predicted to be positive by the model, and TN represents negative samples predicted to be negative by the model. FN represents positive samples predicted to be negative by the model. Removing the above four performance measures, the ROC curve will also be used to assess the effect of our predictions.

### Experimental Results and Analysis

#### Performance of Different Features on Training Set PDB1075

**FIGURE 2:**
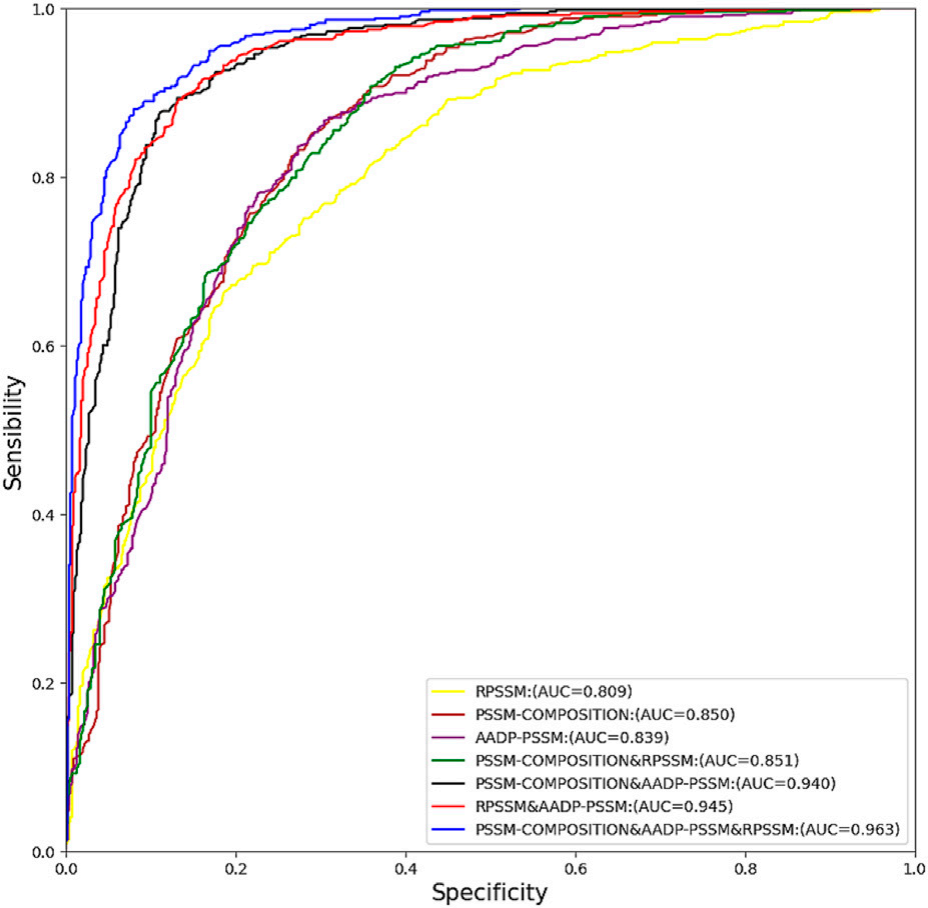
ROC curves with different combinations of features on PDB1075.

A large amount of information on homologous proteins is contained in evolutionarily informative features based on the PSSM matrix. In our method, we selected the evolutionary information-based features PSSM-COMPOSITION, RPSSM, and AADP-PSSM for experimentation. To better show the efficiency of prediction models under different combinations of features, the receiver operating characteristic (ROC) curve was used for analysis. The closer the curve is to the *y*-axis, the better the classification results will be. The area under the curve (AUC) is defined as the area under the ROC curve enclosed by the coordinate axis. The closer the area is to 1, the better the prediction model will be Random forests can achieve better prediction performance when dealing with high-dimensional features. In this section, we use random forests with default hyperparameters on the training set pdb1075 for 10-fold cross validation of different feature fusion schemes and find out the feature fusion method that can maximize the area of AUC. As shown in Figure 2, the prediction performance of RF was the best after fusing the three features, and its AUC area reached 0.963. In addition, we also tested the predictive performance of SVM and KNN under different feature fusion schemes, and their optimal feature fusion schemes had AUC areas of 0.828 and 0.790, respectively. The ROC curve details of SVM and KNN are given in Figure 1 and Figure 2 of supplementary material respectively.

#### Performance After Feature Selection

**FIGURE 3:**
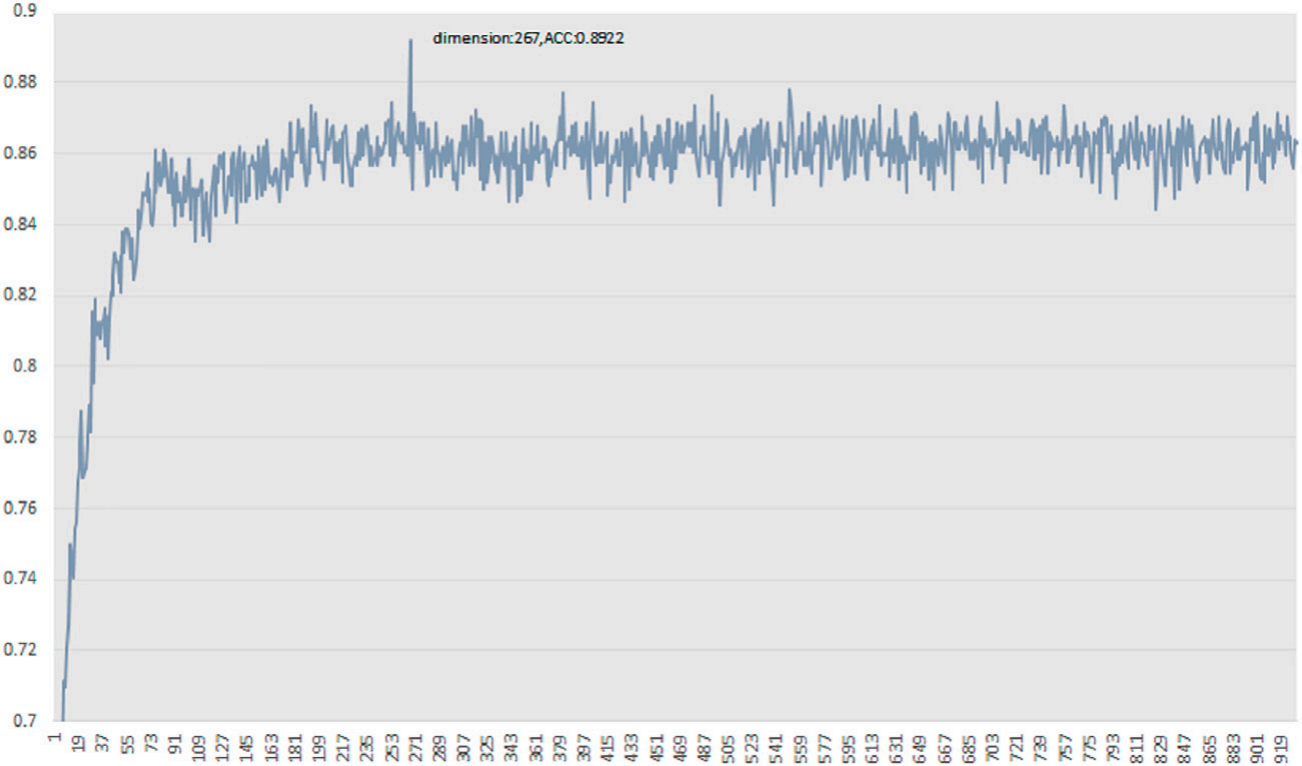
Prediction accuracy curve of feature subset.

For the 930-dimensional features of the initial vector set, we ranked all features from high to low based on MRMD scores. After obtaining the final feature ranking results, we took the first feature as the feature subset and utilised random forest to check the performance of the selected feature subset in 10-fold cross validation on PDB1075. Subsequently, we added one feature in the feature subset, one at a time, according to the feature sorting order. Then we repeated the above process until all the features in the initial feature set were included in the feature subset. Finally, we determined the best predictive accuracy and the optimal feature subset. The results are shown in Figure 3. The feature subset achieves the best accuracy when it contains 267-dimensional features, so the optimal feature subset we used for training models is 267-dimension. The optimal feature subset contains 98-dimensional AADP-PSSM features, 142-dimensional PSSM-COMPOSITION features, and 27-dimensional RPSSM features. The details of the optimal feature subset are given in the supplementary materials. From the distribution of the optimal feature subset, it can be found that the distribution difference of amino acid pairs is the key to identify DBP from massive proteins.

#### Performance of Different Classification Algorithms

**FIGURE 4:**
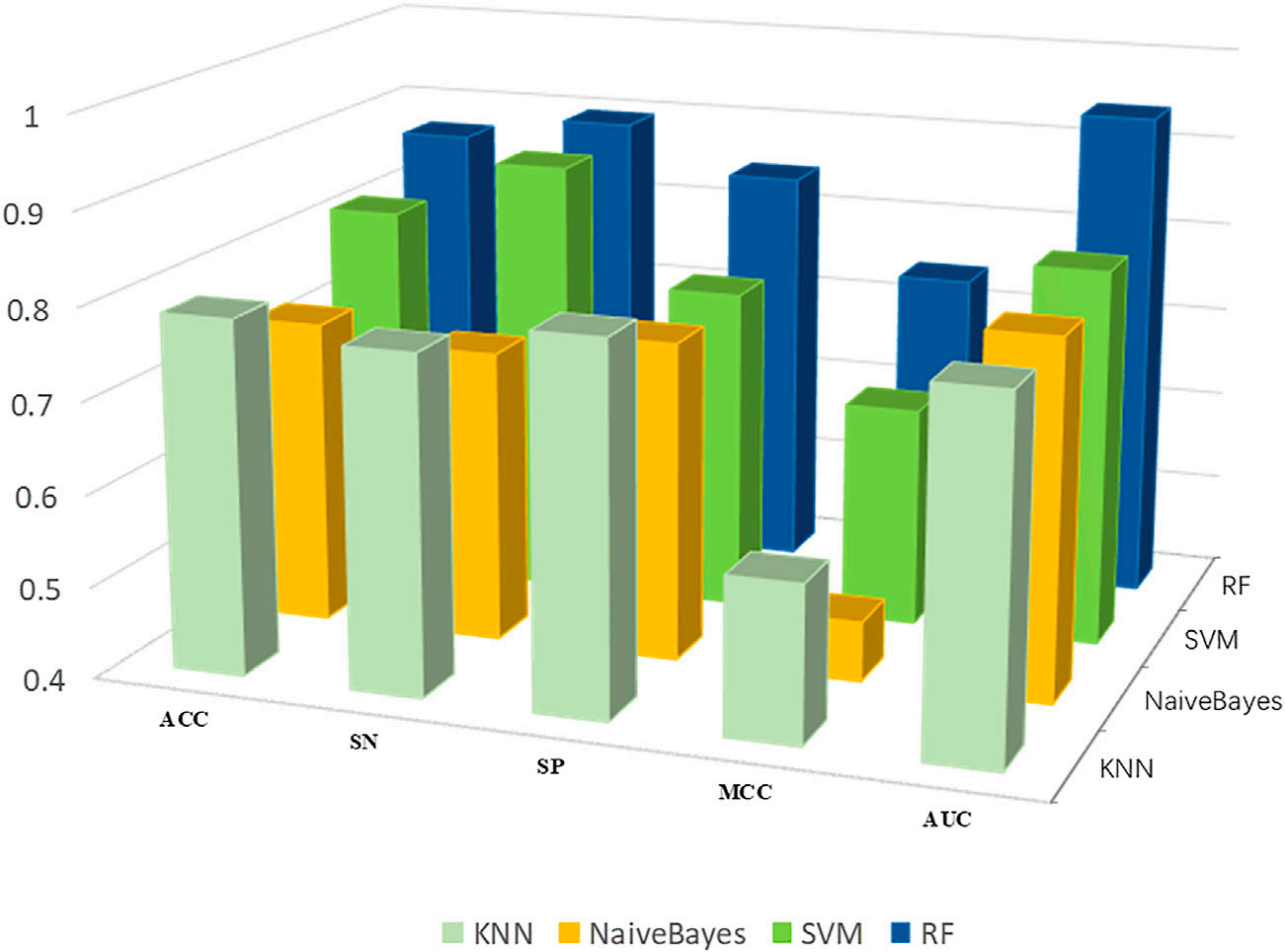
Performance of training set PDB1075 on different classifiers.

To determine the prediction model with the best performance, we put the best feature subset into four powerful classification algorithms with default hyperparameters, KNN, SVM, RF and naïve Bayes, and we used 10-fold cross validation to compare performance. Experimental results show that the random forest method demonstrates the best classification performance (Figure 4).

We use ACC, Sn, SP, MCC and AUC to evaluate the performance. As shown in Figure 4, the five indicators of KNN are 78.6, 76.8, 80.1%, 0.571 and 0.785, respectively. The ACC, Sn, SP, MCC and AUC of SVM were 81.6, 88.2, 75.4%, 0.641 and 0.812, respectively. The ACC, Sn, SP, MCC and AUC of Naïve Bayes were 73.3, 71.8, 74.7%, 0.465 and 0.789, respectively. Finally, the performance of RF in the above evaluation indexes are 86.9, 89.6, 84.5%, 0.741 and 0.941, respectively. The experimental results show that RF can yield better prediction results, which proves that RF is the best classification algorithm for Establishing DNA-binding protein prediction model.

#### Performance of Different Methods on Test Set PDB186

To evaluate the generalization ability of the prediction model proposed in this paper, we tested the model independently using dataset PDB186. Table 2 compares the performance of this study to other prediction methods on the dataset PDB186.

**TABLE 2:** Performance of this method and other existing methods on PDB186.

Methods	ACC (%)	MCC	SN (%)	SP (%)
IDNA-Prot|dis	72.0	0.445	79.5	64.5
DBPPred	76.9	0.538	79.6	74.2
IDNA-Prot	67.2	0.344	67.7	66.7
DNA-Prot	61.8	0.240	69.9	53.8
DNAbinder	60.8	0.216	57.0	64.5
iDNAPro-PseAAC	71.5	0.442	82.8	60.2
Kmer1+ACC	71.0	0.431	82.8	59.1
Local-DPP	79.0	0.625	92.5	65.6
SVM-based method	75.3	0.560	96.8	53.8
KK-DBP	81.2	0.661	97.8	64.5

From Table 2, we can see that on the independent test set PDB186, the ACC, SN, SP of KK-DBP reach 81.2, 97.8 and 64.5%, respectively. In terms of prediction accuracy, KK-DBP is higher than other existing methods. Compared with the current method with the highest accuracy Local-DPP, KK-DBP was improved by 2.2 and 5.3% on the ACC and SN, respectively. SP is slightly lower than Local-DPP and IDNA-Prot. The results of independent verification experiments confirm that KK-DBP has reliable predictive performance and can recognize DBP from a large number of unknown proteins more accurately than existing DBP recognition methods.

## Discussion and Conclusion

A large number of studies have shown that the classification of DNA-binding proteins has important theoretical and practical significance for future genomics and proteomics research. This paper proposes a DNA-binding protein prediction method, called KK-DBP, that is based on multi-feature fusion and improves the feature extraction method in DNA-binding protein prediction. This method uses PSSM features that contain dipeptide composition information for multi-feature fusion to construct the initial feature set, and it obtains the optimal feature subset for modeling by the maximum correlation maximum distance method. Finally, PDB186 was used as an independent test to further evaluate the effectiveness of our method. On the independent test set, the prediction accuracy, sensitivity and specificity of the model reached 81.2, 97.8 and 64.5%, respectively. KK-DBP surpasses existing methods in prediction accuracy, confirming that our method can identify DBP more accurately than existing methods.

Although our method improves the prediction accuracy of DNA-binding proteins, we still do not know how to construct a better feature extraction algorithm based on sequence and structure information. Therefore, our future research direction will be towards finding more distinguishable feature extraction algorithms (Ding et al., 2016; Zeng et al., 2020a; Yang et al., 2021b; Wang et al., 2021d; Jin et al., 2021) and more suitable classifiers (Ding et al., 2019; Ding et al., 2020a; Ding et al., 2020b; Yang et al., 2021c; Guo et al., 2021) and prediction models (Liu et al., 2020b; Zeng et al., 2020b; Chen et al., 2021; Xu et al., 2021c; Song et al., 2021; Xiong et al., 2021) to better recognise DNA-binding proteins.

## Data Availability

The original contributions presented in the study are included in the article/Supplementary Material, further inquiries can be directed to the corresponding authors.

## References

[B1] Altschul S. F. Gish W. Miller W. Myers E. W. Lipman D. J. (1990). Basic Local Alignment Search Tool. J. Mol. Biol. 215 (3), 403–410. 10.1016/s0022-2836(05)80360-2 2231712

[B2] Ao C. Zhou W. Gao L. Dong B. Yu L. (2020). Prediction of Antioxidant Proteins Using Hybrid Feature Representation Method and Random forest. San Diego, CA: Genomics. 10.1016/j.ygeno.2020.08.016 32818637

[B3] Ao C. Zou Q. Yu L. (2021). RFhy-m2G: Identification of RNA N2-Methylguanosine Modification Sites Based on Random forest and Hybrid featuresMethods. San Diego, Calif). 10.1016/j.ymeth.2021.05.016 34033879

[B4] Ao C. Yu L. Zou Q. (2021). Prediction of Bio-Sequence Modifications and the Associations with Diseases. Brief. Funct. genomics 20 (1), 1–18. 10.1093/bfgp/elaa023 33313647

[B5] Chen Y. Ma T. Yang X. Wang J. Song B. Zeng X. (2021). MUFFIN: Multi-Scale Feature Fusion for Drug-Drug Interaction Prediction. Bioinformatics 37, 2651–2658. 10.1093/bioinformatics/btab169 33720331

[B6] Ding S. Li Y. Shi Z. Yan S. (2014). A Protein Structural Classes Prediction Method Based on Predicted Secondary Structure and PSI-BLAST Profile. Biochimie 97, 60–65. 10.1016/j.biochi.2013.09.013 24067326

[B7] Ding Y. Tang J. Guo F. (2019). Identification of Drug-Side Effect Association via Multiple Information Integration with Centered Kernel Alignment. Neurocomputing 325 (24), 211–224. 10.1016/j.neucom.2018.10.028

[B8] Ding Y. Tang J. Guo F. (2020). Identification of Drug-Target Interactions via Dual Laplacian Regularized Least Squares with Multiple Kernel Fusion. Knowledge-Based Syst. 204, 106254. 10.1016/j.knosys.2020.106254

[B9] Ding Y. Tang J. Guo F. (2020). Identification of Drug-Target Interactions via Fuzzy Bipartite Local Model. Neural Comput. Applic 32, 10303–10319. 10.1007/s00521-019-04569-z

[B10] Ding Y. Tang J. Guo F. (2016). Predicting Protein-Protein Interactions via Multivariate Mutual Information of Protein Sequences. Bmc Bioinformatics 17 (1), 398. 10.1186/s12859-016-1253-9 27677692 PMC5039908

[B11] Fu X. Zhu W. Liao B. Cai L. Peng L. Yang J. (2018). Improved DNA-Binding Protein Identification by Incorporating Evolutionary Information into the Chou's PseAAC. IEEE Access, 1.

[B12] Gao M. Skolnick J. Dbd-Hunter (2008). DBD-Hunter: a Knowledge-Based Method for the Prediction of DNA-Protein Interactions. Nucleic Acids Res. 36 (12), 3978–3992. 10.1093/nar/gkn332 18515839 PMC2475642

[B13] Geete K. Pandey M. (2020). Robust Transcription Factor Binding Site Prediction Using Deep Neural Networks. Curr. Bioinformatics 15 (10), 1137–1152.

[B14] Guo X. Zhou W. Shi B. Wang X. Du A. Ding Y. (2021). An Efficient Multiple Kernel Support Vector Regression Model for Assessing Dry Weight of Hemodialysis Patients. Cbio 16 (2), 284–293. 10.2174/1574893615999200614172536

[B15] Guo Z. Wang P. Liu Z. Zhao Y. (2020). Discrimination of Thermophilic Proteins and Non-thermophilic Proteins Using Feature Dimension Reduction. Front. Bioeng. Biotechnol. 8, 584807. 10.3389/fbioe.2020.584807 33195148 PMC7642589

[B16] He S. Guo F. Zou Q. Ding H. (2020). MRMD2.0: A Python Tool for Machine Learning with Feature Ranking and Reduction. Curr. Bioinformatics 15 (10), 1213–1221.

[B17] Jiang Q. Wang G. Jin S. Li Y. Wang Y. (2013). Predicting Human microRNA-Disease Associations Based on Support Vector Machine. Ijdmb 8 (3), 282–293. 10.1504/ijdmb.2013.056078 24417022

[B18] Jin Q. Meng Z. Pham T. D. Chen Q. Wei L. Su R. (2019). DUNet: A Deformable Network for Retinal Vessel Segmentation. Knowledge-Based Syst. 178, 149–162. 10.1016/j.knosys.2019.04.025

[B19] Jin S. Zeng X. Xia F. Huang W. Liu X. (2021). Application of Deep Learning Methods in Biological Networks. Brief. Bioinform. 22 (2), 1902–1917. 10.1093/bib/bbaa043 32363401

[B20] Kim D. E. Chivian D. Baker D. (2004). Protein Structure Prediction and Analysis Using the Robetta Server. Nucleic Acids Res. 32, W526–W531. Web Server issue. 10.1093/nar/gkh468 15215442 PMC441606

[B21] Kumar K. K. Pugalenthi G. Suganthan P. N. (2009). DNA-prot: Identification of DNA Binding Proteins from Protein Sequence Information Using Random forest. J. Biomol. Struct. Dyn. 26 (6), 679–686. 10.1080/07391102.2009.10507281 19385697

[B22] Kumar M. Gromiha M. M. Raghava G. P. (2007). Identification of DNA-Binding Proteins Using Support Vector Machines and Evolutionary Profiles. BMC Bioinformatics 8, 463. 10.1186/1471-2105-8-463 18042272 PMC2216048

[B23] Li J. Pu Y. Tang J. Zou Q. Guo F. (2020). DeepATT: a Hybrid Category Attention Neural Network for Identifying Functional Effects of DNA Sequences. Brief Bioinform 22 (3), bbaa159. 10.1093/bib/bbaa159 32778871

[B24] Li J. Pu Y. Tang J. Zou Q. Guo F. DeepAVP (2020). DeepAVP: A Dual-Channel Deep Neural Network for Identifying Variable-Length Antiviral Peptides. IEEE J. Biomed. Health Inform. 24 (10), 3012–3019. 10.1109/jbhi.2020.2977091 32142462

[B25] Li T. Fan K. Wang J. Wang W. (2003). Reduction of Protein Sequence Complexity by Residue Grouping. Protein Eng. Des. Selection 16 (5), 323–330. 10.1093/protein/gzg044 12826723

[B26] Li T. Li Q.-Z. (2012). Annotating the Protein-RNA Interaction Sites in Proteins Using Evolutionary Information and Protein Backbone Structure. J. Theor. Biol. 312, 55–64. 10.1016/j.jtbi.2012.07.020 22874580

[B27] Liang Z. Y. Lai H. Y. Yang H. Zhang C. J. Yang H. Wei H. H. (2017). Pro54DB: a Database for Experimentally Verified Sigma-54 Promoters. Bioinformatics 33 (3), 467–469. 10.1093/bioinformatics/btw630 28171531

[B28] Lin W.-Z. Fang J.-A. Xiao X. Chou K.-C. (2011). iDNA-Prot: Identification of DNA Binding Proteins Using Random forest with Grey Model. PLoS One 6 (9), e24756. 10.1371/journal.pone.0024756 21935457 PMC3174210

[B29] Liu B. Wang S. Dong Q. Li S. Liu X. (2016). Identification of DNA-Binding Proteins by Combining Auto-Cross Covariance Transformation and Ensemble Learning. IEEE Trans.on Nanobioscience 15 (4), 328–334. 10.1109/tnb.2016.2555951 28113908

[B30] Liu B. Wang S. Wang X. (2015). DNA Binding Protein Identification by Combining Pseudo Amino Acid Composition and Profile-Based Protein Representation. Sci. Rep. 5, 15479. 10.1038/srep15479 26482832 PMC4611492

[B31] Liu B. Xu J. Lan X. Xu R. Zhou J. Wang X. (2014). iDNA-Prot|dis: Identifying DNA-Binding Proteins by Incorporating Amino Acid Distance-Pairs and Reduced Alphabet Profile into the General Pseudo Amino Acid Composition. PloS one 9 (9), e106691. 10.1371/journal.pone.0106691 25184541 PMC4153653

[B32] Liu C. Wei D. Xiang J. Ren F. Huang L. Lang J. (2020). An Improved Anticancer Drug-Response Prediction Based on an Ensemble Method Integrating Matrix Completion and Ridge Regression. Mol. Ther. - Nucleic Acids 21, 676–686. 10.1016/j.omtn.2020.07.003 32759058 PMC7403773

[B33] Liu D. Li G. Zuo Y. (2019). Function Determinants of TET Proteins: the Arrangements of Sequence Motifs with Specific Codes. Brief. Bioinformatics 20 (5), 1826–1835. 10.1093/bib/bby053 29947743

[B34] Liu H. Qiu C. Wang B. Bing P. Tian G. Zhang X. (2021). Evaluating DNA Methylation, Gene Expression, Somatic Mutation, and Their Combinations in Inferring Tumor Tissue-Of-Origin. Front. Cel Dev. Biol. 9, 619330. 10.3389/fcell.2021.619330 PMC8126648 34012960

[B35] Liu J. Lian X. Liu F. Yan X. Cheng C. Cheng L. (2020). Identification of Novel Key Targets and Candidate Drugs in Oral Squamous Cell Carcinoma. Cbio 15 (4), 328–337. 10.2174/1574893614666191127101836

[B36] Liu T. Zheng X. Wang J. (2010). Prediction of Protein Structural Class for Low-Similarity Sequences Using Support Vector Machine and PSI-BLAST Profile. Biochimie 92 (10), 1330–1334. 10.1016/j.biochi.2010.06.013 20600567

[B37] Long J. Yang H. Yang Z. Jia Q. Liu L. Kong L. (2021). Integrated Biomarker Profiling of the Metabolome Associated with Impaired Fasting Glucose and Type 2 Diabetes Mellitus in Large-Scale Chinese Patients. Clin. Transl Med. 11 (6), e432. 10.1002/ctm2.432 34185410 PMC8167862

[B38] Lu W. Song Z. Ding Y. Wu H. Cao Y. Zhang Y. (2020). Use Chou's 5-Step Rule to Predict DNA-Binding Proteins with Evolutionary Information. Biomed. Res. Int. 2020, 6984045. 10.1155/2020/6984045 32775434 PMC7407024

[B39] Lv H. Dao F. Y. Zulfiqar H. Lin H. (2021). DeepIPs: Comprehensive Assessment and Computational Identification of Phosphorylation Sites of SARS-CoV-2 Infection Using a Deep Learning-Based Approach. Brief. Bioinformatics 22 (6). bbab244. 10.1093/bib/bbab244 34184738 PMC8406875

[B40] Lv H. Dao F. Y. Zulfiqar H. Su W. Ding H. Liu L. (2021). A Sequence-Based Deep Learning Approach to Predict CTCF-Mediated Chromatin Loop. Brief. Bioinformatics 22 (5), bbab031. 10.1093/bib/bbab031 33634313

[B41] Manavalan B. Basith S. Shin T. H. Wei L. Lee G. (2019). mAHTPred: a Sequence-Based Meta-Predictor for Improving the Prediction of Anti-hypertensive Peptides Using Effective Feature Representation. Bioinformatics 35 (16), 2757–2765. 10.1093/bioinformatics/bty1047 30590410

[B42] Manavalan B. Basith S. Shin T. H. Wei L. Lee G. (2019). Meta-4mCpred: A Sequence-Based Meta-Predictor for Accurate DNA 4mC Site Prediction Using Effective Feature Representation. Mol. Ther. - Nucleic Acids 16, 733–744. 10.1016/j.omtn.2019.04.019 31146255 PMC6540332

[B43] Meng F. Kurgan L. (2016). DFLpred: High-Throughput Prediction of Disordered Flexible Linker Regions in Protein Sequences. Bioinformatics 32 (12), i341–i350. 10.1093/bioinformatics/btw280 27307636 PMC4908364

[B44] Qu K. Wei L. Zou Q. (2019). A Review of DNA-Binding Proteins Prediction Methods. Cbio 14 (3), 246–254. 10.2174/1574893614666181212102030

[B45] Shen Z. Zou Q. (2020). Basic Polar and Hydrophobic Properties Are the Main Characteristics that Affect the Binding of Transcription Factors to Methylation Sites. Bioinformatics 36 (15), 4263–4268. 10.1093/bioinformatics/btaa492 32399547

[B46] Song B. Huang S. Zeng X. (2021). The Computational Power of Monodirectional Tissue P Systems with Symport Rules. Inf. Comput., 104751. 10.1016/j.ic.2021.104751

[B47] Stawiski E. W. Gregoret L. M. Mandel-Gutfreund Y. (2003). Annotating Nucleic Acid-Binding Function Based on Protein Structure. J. Mol. Biol. 326 (4), 1065–1079. 10.1016/s0022-2836(03)00031-7 12589754

[B48] Su R. Liu X. Wei L. Zou Q. (2019). Deep-Resp-Forest: A Deep forest Model to Predict Anti-cancer Drug Response. Methods 166, 91–102. 10.1016/j.ymeth.2019.02.009 30772464

[B49] Su W. Liu M.-L. Yang Y.-H. Wang J.-S. Li S.-H. Lv H. (2021). PPD: A Manually Curated Database for Experimentally Verified Prokaryotic Promoters. J. Mol. Biol. 433 (11), 166860. 10.1016/j.jmb.2021.166860 33539888

[B50] Tang X. Cai L. Meng Y. Gu C. Yang J. Yang J. (2021). A Novel Hybrid Feature Selection and Ensemble Learning Framework for Unbalanced Cancer Data Diagnosis with Transcriptome and Functional Proteomic. IEEE Access 9, 51659–51668. 10.1109/access.2021.3070428

[B51] Tao Z. Li Y. Teng Z. Zhao Y. (2020). A Method for Identifying Vesicle Transport Proteins Based on LibSVM and MRMD. Comput. Math. Methods Med. 2020, 8926750. 10.1155/2020/8926750 33133228 PMC7591939

[B52] Wang D. Zhang Z. Jiang Y. Mao Z. Wang D. Lin H. (2021). DM3Loc: Multi-Label mRNA Subcellular Localization Prediction and Analysis Based on Multi-Head Self-Attention Mechanism. Nucleic Acids Res. 49 (8), e46. 10.1093/nar/gkab016 33503258 PMC8096227

[B53] Wang H. Ding Y. Tang J. Zou Q. Guo F. (2021). Identify RNA-Associated Subcellular Localizations Based on Multi-Label Learning Using Chou's 5-steps Rule. BMC Genomics 22 (1), 56. 10.1186/s12864-020-07347-7 33451286 PMC7811227

[B54] Wang X. Yang Y. Liu J. Wang G. (2021). The Stacking Strategy-Based Hybrid Framework for Identifying Non-coding RNAs. Brief Bioinform 22 (5), bbab023. 10.1093/bib/bbab023 33693454

[B55] Wang Z. Liu D. Xu B. Tian R. Zuo Y. (2021). Modular Arrangements of Sequence Motifs Determine the Functional Diversity of KDM Proteins. Brief Bioinform 22 (3). 10.1093/bib/bbaa215 32987405

[B56] Wei L. Liao M. Gao Y. Ji R. He Z. Zou Q. (2014). Improved and Promising Identification of Human MicroRNAs by Incorporating a High-Quality Negative Set. Ieee/acm Trans. Comput. Biol. Bioinf. 11 (1), 192–201. 10.1109/tcbb.2013.146 26355518

[B57] Wei L. Tang J. Zou Q. (2017). Local-DPP: An Improved DNA-Binding Protein Prediction Method by Exploring Local Evolutionary Information. Inf. Sci. 384, 135–144. 10.1016/j.ins.2016.06.026

[B58] Wei L. Xing P. Zeng J. Chen J. Su R. Guo F. (2017). Improved Prediction of Protein-Protein Interactions Using Novel Negative Samples, Features, and an Ensemble Classifier. Artif. Intelligence Med. 83, 67–74. 10.1016/j.artmed.2017.03.001 28320624

[B59] Wu X. Yu L. (2021). EPSOL: Sequence-Based Protein Solubility Prediction Using Multidimensional Embedding. Oxford, England: Bioinformatics. 10.1093/bioinformatics/btab463 34145885

[B60] Xiong G. Wu Z. Yi J. Fu L. Yang Z. Hsieh C. (2021). ADMETlab 2.0: an Integrated Online Platform for Accurate and Comprehensive Predictions of ADMET Properties. Nucleic Acids Res. 49 (W1), W5–W14. 10.1093/nar/gkab255 33893803 PMC8262709

[B61] Xu B. Liu D. Wang Z. Tian R. Zuo Y. (2021). Multi-substrate Selectivity Based on Key Loops and Non-homologous Domains: New Insight into ALKBH Family. Cell. Mol. Life Sci. 78 (1), 129–141. 10.1007/s00018-020-03594-9 32642789 PMC11072825

[B62] Xu H. Zeng W. Zeng X. Yen G. G. (2021). A Polar-Metric-Based Evolutionary Algorithm. IEEE Trans. Cybern. 51, 3429–3440. 10.1109/TCYB.2020.2965230 32031958

[B63] Xu L. Jiang S. Wu J. Zou Q. (2021). An In Silico Approach to Identification, Categorization and Prediction of Nucleic Acid Binding Proteins. Brief Bioinform 22 (3), bbaa171. 10.1093/bib/bbaa171 32793956

[B64] Yang C. Ding Y. Meng Q. Tang J. Guo F. (2021). Granular Multiple Kernel Learning for Identifying RNA-Binding Protein Residues via Integrating Sequence and Structure Information. Neural Comput. Appl. 33, 11387–11399. 10.1007/s00521-020-05573-4

[B65] Yang H. Ding Y. Tang J. Guo F. (2021). Drug-disease Associations Prediction via Multiple Kernel-Based Dual Graph Regularized Least Squares. Appl. Soft Comput. 112, 107811. 10.1016/j.asoc.2021.107811

[B66] Yang H. Luo Y. Ren X. Wu M. He X. Peng B. (2021). Risk Prediction of Diabetes: Big Data Mining with Fusion of Multifarious Physical Examination Indicators. Inf. Fusion 75, 140–149. 10.1016/j.inffus.2021.02.015

[B67] Yu L. Xu F. Gao L. (2020). Predict New Therapeutic Drugs for Hepatocellular Carcinoma Based on Gene Mutation and Expression. Front. Bioeng. Biotechnol. 8, 8. 10.3389/fbioe.2020.00008 32047745 PMC6997129

[B68] Zeng X. Wang W. Chen C. Yen G. G. (2020). A Consensus Community-Based Particle Swarm Optimization for Dynamic Community Detection. IEEE Trans. Cybern. 50 (6), 2502–2513. 10.1109/tcyb.2019.2938895 31545758

[B69] Zeng X. Zhu S. Hou Y. Zhang P. Li L. Li J. (2020). Network-based Prediction of Drug-Target Interactions Using an Arbitrary-Order Proximity Embedded Deep forest. Bioinformatics 36 (9), 2805–2812. 10.1093/bioinformatics/btaa010 31971579 PMC7203727

[B70] Zhai Y. Chen Y. Teng Z. Zhao Y. (2020). Identifying Antioxidant Proteins by Using Amino Acid Composition and Protein-Protein Interactions. Front. Cel Dev. Biol. 8, 591487. 10.3389/fcell.2020.591487 PMC7658297 33195258

[B71] Zhang D. Chen H. D. Zulfiqar H. Yuan S. S. Huang Q. L. Zhang Z. Y. (2021). iBLP: An XGBoost-Based Predictor for Identifying Bioluminescent Proteins. Comput. Math. Methods Med. 2021, 6664362. 10.1155/2021/6664362 33505515 PMC7808816

[B72] Zhang D. Xu Z. C. Su W. Yang Y. H. Lv H. Yang H. (2020). iCarPS: a Computational Tool for Identifying Protein Carbonylation Sites by Novel Encoded Features. Bioinformatics 37 (2), 171–177. 10.1093/bioinformatics/btaa702 32766811

[B73] Zhang J. Zhang Z. Pu L. Tang J. Guo F. (2020). AIEpred: an Ensemble Predictive Model of Classifier Chain to Identify Anti-inflammatory Peptides. Ieee/acm Trans. Comput. Biol. Bioinform, 1. 10.1109/TCBB.2020.2968419 31985437

[B74] Zhao X. Jiao Q. Li H. Wu Y. Wang H. Huang S. (2020). ECFS-DEA: an Ensemble Classifier-Based Feature Selection for Differential Expression Analysis on Expression Profiles. BMC Bioinformatics 21 (1), 43. 10.1186/s12859-020-3388-y 32024464 PMC7003361

[B75] Zhao X. Wang H. Li H. Wu Y. Wang G. (2021). Identifying Plant Pentatricopeptide Repeat Proteins Using a Variable Selection Method. Front. Plant Sci. 12, 506681. 10.3389/fpls.2021.506681 33732270 PMC7957076

[B76] Zheng L. Huang S. Mu N. Zhang H. Zhang J. Chang Y. (2019). RAACBook: a Web Server of Reduced Amino Acid Alphabet for Sequence-dependent Inference by Using Chou's Five-step Rule. Database (Oxford) 2019, baz131. 10.1093/database/baz131 31802128 PMC6893003

[B77] Zhu Y. Li F. Xiang D. Akutsu T. Song J. Jia C. (2021). Computational Identification of Eukaryotic Promoters Based on Cascaded Deep Capsule Neural Networks. Brief Bioinform 22 (4). 10.1093/bib/bbaa299 PMC8522485 33227813

[B78] Zou L. Nan C. Hu F. (2013). Accurate Prediction of Bacterial Type IV Secreted Effectors Using Amino Acid Composition and PSSM Profiles. Bioinformatics 29 (24), 3135–3142. 10.1093/bioinformatics/btt554 24064423 PMC5994942

[B79] Zou Q. Zeng J. Cao L. Ji R. (2016). A Novel Features Ranking Metric with Application to Scalable Visual and Bioinformatics Data Classification. Neurocomputing 173, 346–354. 10.1016/j.neucom.2014.12.123

[B80] Zou Y. Wu H. Guo X. Peng L. Ding Y. Tang J. (2021). MK-FSVM-SVDD: A Multiple Kernel-Based Fuzzy SVM Model for Predicting DNA-Binding Proteins via Support Vector Data Description. Cbio 16 (2), 274–283. 10.2174/1574893615999200607173829

[B81] Zulfiqar H. Yuan S.-S. Huang Q.-L. Sun Z.-J. Dao F.-Y. Yu X.-L. (2021). Identification of Cyclin Protein Using Gradient Boost Decision Tree Algorithm. Comput. Struct. Biotechnol. J. 19, 4123–4131. 10.1016/j.csbj.2021.07.013 34527186 PMC8346528

[B82] Zuo Y.-C. Peng Y. Liu L. Chen W. Yang L. Fan G.-L. (2014). Predicting Peroxidase Subcellular Location by Hybridizing Different Descriptors of Chou' Pseudo Amino Acid Patterns. Anal. Biochem. 458, 14–19. 10.1016/j.ab.2014.04.032 24802134

[B83] Zuo Y. Li Y. Chen Y. Li G. Yan Z. Yang L. (2017). PseKRAAC: a Flexible Web Server for Generating Pseudo K-Tuple Reduced Amino Acids Composition. Bioinformatics 33 (1), 122–124. 10.1093/bioinformatics/btw564 27565583

