# Supplementary material for: KK-DBP: A Multi-Feature Fusion Method for DNA-Binding Protein Identification Based on Random Forest
Source: Front Genet. 2021 Nov 29;12:811158. doi: 10.3389/fgene.2021.811158 (PMC8667860; doi:10.3389/fgene.2021.811158)
Supplement: Supplementary file 1 [file DataSheet1.ZIP › Supplementary materials/Supplementary materials .docx]

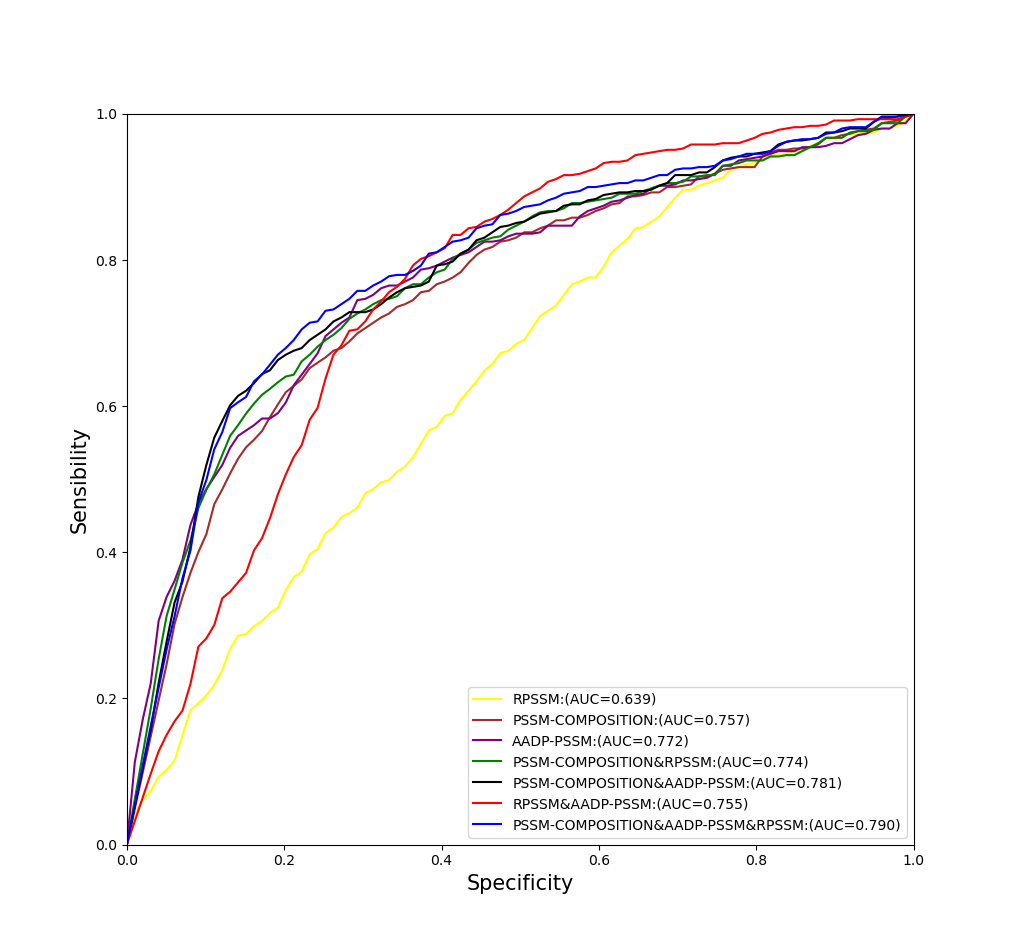


Supplementary Figure 1：ROC curves with different combinations of features on pdb1075 using KNN
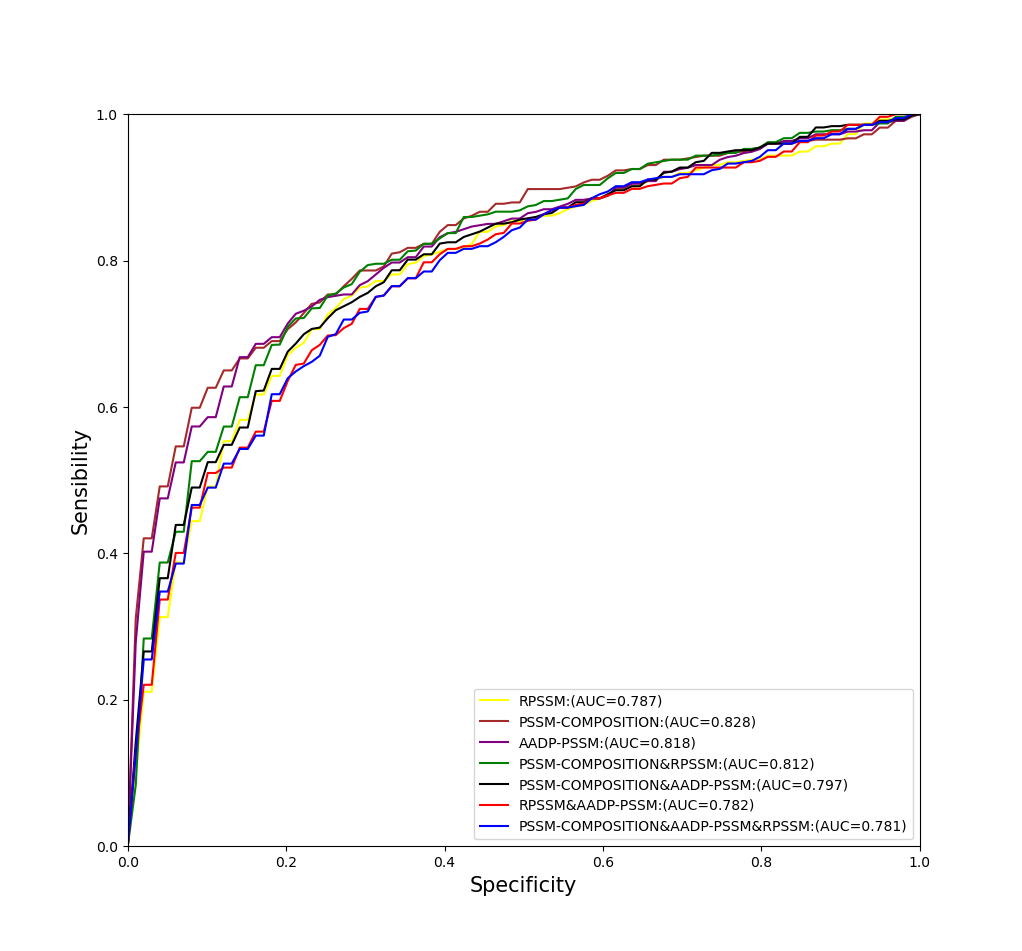
 Supplementary Figure 1：ROC curves with different combinations of features on pdb1075 using SVM
